# Soluble CD163 does not predict first-time myocardial infarction in patients infected with human immunodeficiency virus: a nested case–control study

**DOI:** 10.1186/1471-2334-13-230

**Published:** 2013-05-21

**Authors:** Andreas Knudsen, Holger Jon Møller, Terese L Katzenstein, Jan Gerstoft, Niels Obel, Gitte Kronborg, Thomas Benfield, Andreas Kjaer, Anne-Mette Lebech

**Affiliations:** 1 Department of Infectious Diseases, Copenhagen University Hospital, Kettegaard Allé 30, Hvidovre 2650, Denmark; 2 Department of Clinical Physiology, Nuclear Medicine & PET, Copenhagen University Hospital, Rigshospitalet, Copenhagen, Denmark; 3 Department of Clinical Biochemistry, Aarhus University Hospital, Aarhus, Denmark; 4 Department of Infectious Diseases, Copenhagen University Hospital, Rigshospitalet, Copenhagen, Denmark

**Keywords:** HIV-infection, Myocardial infarction, Soluble CD163

## Abstract

**Background:**

Soluble CD163 (sCD163) has been associated with arterial inflammation and non-calcified plaques in human immunodeficiency virus (HIV)-infected individuals and has therefore been suggested as a predictive biomarker of myocardial infarction (MI).

**Methods:**

We conducted a nested case–control study of 55 cases with first-time MI and 182 controls matched for age, duration of antiretroviral therapy (ART), gender, smoking, and no known cardiovascular disease. All patients had four available plasma samples, 1: Before initiation of antiretroviral therapy (ART), 2: Three months after ART, 3: One year before the case’s MI, and 4: The last sample available before the case’s MI. We used conditional logistic regression to estimate the association of sCD163 with first-time MI.

**Results:**

The two groups had similar HIV-parameters and cardiovascular risk factors were equally distributed. There was no significant association between sCD163 and MI neither in samples obtained one year before (OR 1.05, CI 95% 0.85 – 1.29, *p* = 0.66) nor two months before (OR 1.20, CI 95% 0.98-1.47 *p* = 0.08).

**Conclusion:**

sCD163 did not prove to be a useful biomarker for prediction of first-time MI in a HIV-infected population.

## Background

Patients infected with human immunodeficiency virus (HIV) are at increased risk of myocardial infarction (MI) [1], but the pathophysiological mechanisms are not fully understood.

Studies have found not only a higher prevalence of traditional risk factors for cardiovascular disease (CVD) [2] but also a high prevalence of asymptomatic atherosclerotic lesions among HIV-infected individuals [3]. These lesions are mainly non-calcified and have higher contents of inflammatory components, which make them more vulnerable and prone to rupture [4].

Soluble CD163 (sCD163) is a marker of activated macrophages and is associated with coronary atherosclerotic burden in the general population [5] and both arterial inflammation and non-calcified plaques in HIV-infected individuals [6,7]. Therefore, we sought to investigate if sCD163 may serve as a prognostic marker of first-time MI in an HIV-infected population. We analyzed levels of sCD163 in plasma from 55 HIV-infected patients with first-time MI at four time points and compared them to matched HIV-infected patients without cardiovascular events.

## Methods

We conducted a nested case–control study by extracting data on patients given a diagnosis of ischaemic heart disease and HIV (International Classification of Diseases-10th Revision (ICD-10) I20-25, and HIV, ICD-10 B20-24) from January 1998 to December 2008. Data were extracted from the Danish National Hospital Register, which records data on all patients discharged from a non-psychiatric hospital in Denmark. We then identified the patients registered in the Danish HIV Cohort Study (DHCS), which is a nationwide, prospective, population-based cohort study of all Danish HIV-infected individuals described elsewhere [8]. Among these we identified patients followed at either Department of Infectious diseases, Rigshospitalet or Department of Infectious Dieases, Hvidovre Hospital, Copenhagen, Denmark, which have systematically saved plasma samples from all quarterly visits by HIV-infected patients. All patients had given written consent to further analysis of the blood samples. We then conferred with patients’ medical records and only patients with verified MI according to international criteria [9] were included in the study.

The date of MI served as index date for the selection of controls, who were already enrolled in the DHCS at the time of case’s MI. We used an incidence density sampling and obtained up to 4 controls per case. We excluded cases and controls with diabetes and/or prior cardiovascular disease, other than hypertension. All patients in the study received antiretroviral therapy (ART).

The study was approved by the scientific ethics committee of the capital region of Denmark (reference number H-D-2008-108).

### Matching

Controls were matched with their case for age at the time of MI ± 3 years, gender, duration of ART, and smoking with a dichotomous status of either *smoking* (ever) or *non-smoking* (never).

### Study set-up

The study set-up included 4 time points for each patient where both plasma samples had been frozen and quarterly blood analysis had been performed: Sample 1: last sample before initiation of ART, Sample 2: three months after initiation of ART, Sample 3: one year before case’s MI/index date, and Sample 4: last sample before case’s MI/index date. Time intervals between the samples are shown in Table 1.

**Table 1:** Demographic, metabolic and HIV characteristics of the patients

**Variable**	**Cases**	**Controls**	***p*-value**^**a**^
Number of patients	55	182	
Gender (male/female) (%)	50/5 (91/9)	167/15 (92/8)	
Age at time of MI/index date median (IQR), years	49 (42 – 57)	50 (43 – 57)	
Duration of HIV before MI/index date median (IQR), years	10 (6 – 17)	10 (7 – 16)	0.71
Mean duration of therapy before MI/index date median (IQR), years	6 (3 – 8)	6 (3 – 9)	
Intervals between plasma samples			
From sample 1 to initiation ART, median (IQR), days	42 (24 – 76)	49 (32 – 81)	
From initiation of ART to sample 2 median (IQR), days	99 (88 – 123)	109 (87 – 142)	
From sample 3 to MI/index date median (IQR), days	334 (292 – 367)	368 (334 – 408)	
From sample 4 to MI/index date median (IQR), days	52 (27 – 82)	0 (0 – 0)	
Smoking (never/ever) (%)	2/53 (4/96)	6/176 (3/97)	
Blood pressure, systolic median (IQR)	135 (120 – 149) n = 36	125 (115 – 140) n = 87	0.09
CD4 cell count/mm^3^, median (IQR)^¤^	496 (290–688) n = 52	547 (307–800) n = 161	0.78
HIV-RNA copies/mL, median (range)^¤^	39 (19–217200)	39 (19–93900)	0.08
Number of patients with HIV-RNA < 400 copies/mL^¤^	44 (80%)	146 (89%)	0.12
Creatinine μM, median (IQR)^¤^	81 (70–89)	78 (70–88)	0.38
mg/dL, median (IQR)	1.1 (0.9–1.2) n = 48	1.0 (0.9–1.2) n = 148	
Cholesterol mM, median (IQR)^¤^	6.3 (5.5–8.0)	5.7 (5.0–6.7)	0.38
mg/dL, median (IQR)	241 (214–309) n = 32	220 (193–255) n = 96	
Lipid-lowering treatment (%)^#^	6	4	0.6
Starting antihypertensive treatment (%)^#^	13	4	0.07
Starting anti-coagulative treatment (%)^#^	4	3	0.67
Co-infected with hepatitis B (%) (HbsAg positive)	6	7	0.6
Co-infected with hepatitis C (%) (HCV-Ab-positive)	6	14	0.07
Exposed to ART (%)^#^	100	100	-
Exposed to NRTI (%)^#^	100	99.5	0.76
Exposed to abacavir (%)^#^	56	39	0.007
Exposed to NNRTI (%)^#^	73	58	0.006
Exposed to PI (%)^#^	87	84	0.24

^a^ univariate conditional logistic regression.

^¤^ plasma sample 4 (at time of MI/index date).

^#^ within the study period.

Abbreviations: *ART*, Antiretroviral therapy; B-glucose, blood-glucose; *HIV*, Human immunodeficiency virus; *MI*, Myocardial infarction; *NNRTI*, Nonnucleoside reverse-transcriptase inhibitors; *NRTI*, Nucleoside reverse-transcriptase inhibitors; *PI*, Protease inhibitors.

### Risk estimation

An individual risk estimation was calculated based on blood pressure measurements within the study period and values of total cholesterol from sample 3 or 4 using the HeartScore for European high risk countries that Denmark belonged to at time of cases’ MI. This model estimates the 10-year risk of fatal CVD [10].

### Plasma analysis of CD163

Plasma samples were all stored at −80°C until the analysis of sCD163, which was determined in duplicate by an in-house sandwich enzyme-linked immunosorbent assay using a BEP-2000 ELISA-analyser (Dade Behring, Deerfield, IL, USA) essentially as previously described [11]. Briefly, rabbit anti-CD163 (2 mg/L) was coated onto microtitre wells and plates transferred to a BEP-2000 enzyme-linked immunosorbent assay (ELISA)-analyzer (Dade Behring, Eschborn, Germany). Samples (diluted 1:101) were added in duplicates and incubated for 1.5 h at 37°C. Monoclonal anti-CD163 (GHI/61, 3 μg/mL) was added followed by incubation for 1 h at 37°C with horseradish peroxidase-labelled goat antimouse antibodies (0.125 μg/mL; Dako, Glostrup, Denmark). The assay was calibrated using serum traceable to purified human CD163, with the lowest calibrator being 6.25 μg/L. The inter-assay coefficient of variation was 3.0% at 1.33 mg/L and 4.7% at 3.71 mg/L on control samples included on each plate (28 runs). sCD163 is stable in plasma and withstands repeated freeze-thaw cycles of samples [12].

### Statistical analysis

We used conditional logistic regression to estimate odds ratios (OR) and 95% confidence intervals (CI) for the association of sCD163 and MI and for comparison between cases and controls. We adjusted for covariables by performing multiple conditional regression analysis. sCD163 was used as a continuous variable.

Levels of sCD163 at different time points within the groups were compared using a paired *t*-test on log_10_-transformed data. We compared differences in levels of log_10_-transformed sCD163 according to treatment regimen with an independent samples *t*-test.

The prognostic performance of sCD163 was evaluated constructing a reciever operating characteristic (ROC) curve. With a 55 cases and 182 controls the study was powered at 80% with a 2-sided significance level of 0.05 to detect an absolute difference of 0.5 mg/L sCD163 based on previously reported standard deviation. We considered a *p* < 0.05 significant. All statistics were performed on SPSS 20 (IBM SPSS Statistics for Windows, Version 20.0. Armonk, NY: IBM Corp.).

## Results

We identified 55 cases and 182 controls. Twenty seven cases had 4 controls, 20 cases had 3 controls, 6 cases had 2 and 2 cases had 1 control. Patient characteristics are shown in Table 1.

Both cases and controls were predominantly white (93 and 94% respectively). Modes of infection were similar between the groups with 69 and 67% homosexually infected, 18 and 22% heterosexually infected, and 4 and 6% infected by intravenous drug abuse respectively. The 10-year risk of fatal CVD was calculated in the 99 subjects who had all the necessary parameters (29 cases and 70 controls) and there was no significant difference between the two groups (3.3 in the control group and 5.3 among cases, *p* = 0.20).

More than 80% of the patients in both groups had suppressed viral load (< 400 copies/mL) and CD4 cell counts above 400 cells/μL in plasma sample 4, and metabolic cardiovascular risk factors were equally distributed in cases and controls. A total of 32 cases had ST-elevation MI (STEMI), 16 cases had Non-ST-elevation MI (NSTEMI) and 7 cases had non-specified MI.

The initiation of ART resulted in a significant increase in CD4 cell counts and a significant decrease in HIV RNA levels in both groups. The suppression of viral load was maintained equally in both groups, and CD4 cells continued to rise in the following samples.

**Figure 1:**
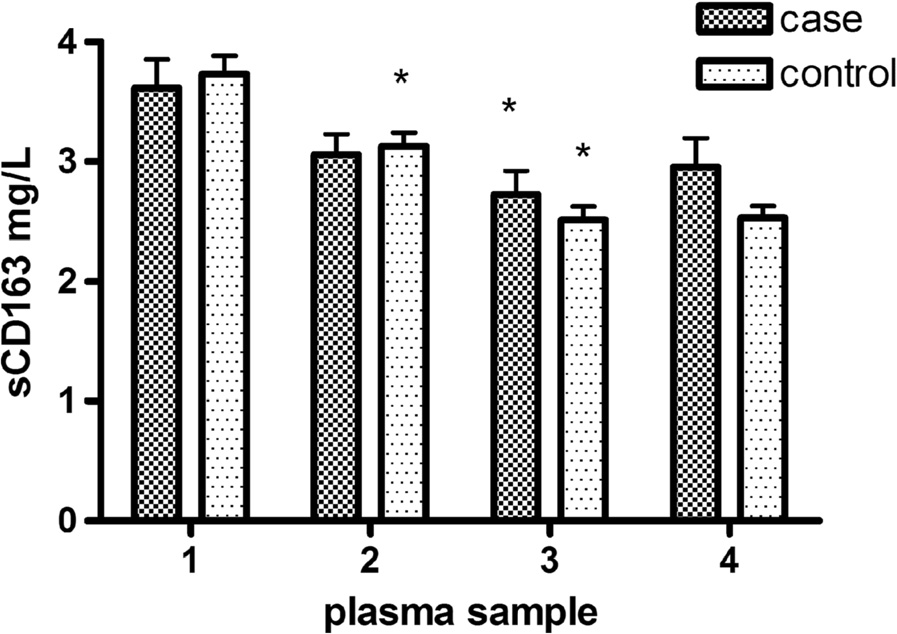
**Mean plasma levels of sCD163 (mg/L) at 1:last sample before initiation of ART, 2: three months after ART, 3: one year before case’s MI/index date, and 4: last sample before case’s MI/index date.** Bars represent standard error of the mean (SEM). * Indicates a significant decrease in plasma level of sCD163 from the previous time point (p < 0.05).

Figure 1 shows that both groups decreased in plasma levels of sCD163 3 months after initiation of ART although only the control group reached significance. The plasma levels continued to decrease during ART reaching steady levels 1 year prior to the case’s MI. The cases showed no statistically significant rise in sCD163 plasma levels prior to their MI.

The two groups did not differ in plasma levels of sCD163 in any of the 4 consecutive samples, but the association between sCD163 and MI grew stronger in sample 4 (OR 1.2, CI 0.98-1.47, *p* = 0.08), Table 2.

**Table 2:** Median plasma levels of sCD163 in mg/L (IQR) at 1: last sample before initiation of ART, 2: three months after ART, 3: one year before case’s MI/index date, and 4: last sample before case’s MI/index date

	**Sample 1**	**Sample 2**	**Sample 3**	**Sample 4**
Cases	3.22	2.87	2.33	2.38
	(2.53 – 4.32)	(2.22 – 3.51)	(1.73 – 3.31)	(1.86 – 3.58)
	n = 51	n = 49	n = 51	n = 55
Controls	3.03	2.68	2.24	2.24
	(2.50 – 4.58)	(2.13 – 3.70)	(1.65 – 2.98)	(1.69 – 2.99)
	n = 165	n = 161	n = 172	n = 176
*p* – values^a^	0.67	0.48	0.66	0.08

^a^ univariate conditional logistic regression.

**Figure 2:**
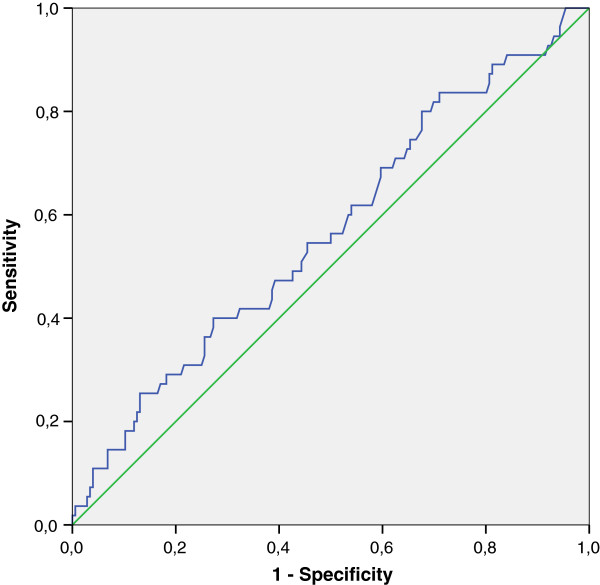
**Receiver-operator characteristics (ROC) curve for plasma sCD163 at sample 4 for the occurrence of MI.** Area under the curve (AUC) for sCD163 is 0.57 (CI 95% 0.48-0.65 *p* = 0.14).

In a multiple conditional regression model adjusting for HIV RNA sCD163 remained non significant (OR 1.20, CI 0.96 – 1.51, *p* = 0.11) To evaluate the possible role of sCD163 in sample 4 as a predictor of MI we constructed a ROC curve for sCD163 which gave an area under the curve of 0.57 (CI 95% 0.48-0.65 *p* = 0.14), Figure 2.

All patients in the study received ART and had all been exposed to nucleosid reverse transcriptase inhibitors (NRTIs) and both groups had been equally exposed to protease inhibitors (PIs) (*p* = 0.24). A significantly larger number of cases had been exposed to NNRTIs (*p* = 0.006) and the NRTI, abacavir (*p* = 0.007). In a multiple conditional regression model adjusting for exposure to abacavir, NNRTI, and PI sCD163 remained non significant (OR 1.14, CI 0.92 – 1.40, *p* = 0.22).

No difference in plasma levels of sCD163 was found between cases exposed (n = 31) and not exposed (n = 24) to abacavir in plasma sample 3 or 4 (*p* = 0.71 and 0.20 respectively).

## Discussion

In this case–control study of 237 HIV-infected patients with similar cardiovascular risk factors and comparable risk estimations we did not find that sCD163 could serve as predictive biomarker of first-time MI. Macrophages are key players in the development of atherosclerosis [13] and their possible mechanistic role behind the increased risk in the HIV-infected population is currently subject to attention [14]. Studies in HIV-infected patients have recently associated markers of activated macrophages with arterial inflammation [7], increased progression of carotid intima-media thickness [15], and noncalcified plaques [6] suggesting that these markers may serve as predictors of clinical events.

Despite these promising results, our study did not support the use of sCD163 as a predictive marker of first-time MI in an HIV-infected population neither one year before MI nor approximately 2 months before MI as underlined by the ROC-curve in Figure 2.

A recent study found an association between HIV-RNA levels >50 copies/mL and MI in patients infected with HIV [16], and HIV-infection has, primarily through HIV-RNA viral load, been shown to increase markers of endothelial dysfunction [17]. We therefore performed an analysis of the association of sCD163 and MI adjusting for viral load which remained non-significant.

The association between abacavir and MI has been thoroughly examined in the recent years with different conclusions [18]. Data from the SMART-study and D:A:D-study group suggested that abacavir had proinflammatory properties causing elevated levels of high sensitivity C-reactive protein and interleukin 6, and that the increased risk of MI therefore could relay on vascular inflammation [19]. In a study with positron emission tomography sCD163 has recently been associated with vascular inflammation [7], but our model adjusting for antiretroviral treatment did not, however, suggest that abacavir influenced the levels of sCD163 and levels of sCD163 were not significantly higher in cases treated with abacavir. This suggests a different mechanism of action other than activation of macrophages if abacavir is contributing to the risk of MI.

To the best of our knowledge this study is the first to evaluate the association of sCD163 and MI in a population infected with HIV. This nested case–control setup provides unique possibility to assess the association between sCD163 and hard end-point cardiovascular disease in a cohort of well controlled and closely followed patients infected with HIV who had blood samples analysed quarterly at university centres. We matched our patients for factors known to influence the risk of MI thus focusing on sCD163.

On the other hand, case–control studies do offer certain challenges in form of e.g. selection bias, which we tried to account for since all cases were possible controls until their MI. The cases were selected from a period of ten years and laboratory techniques may have changed over time and some analyses were not available during the first years. Some samples had been frozen for several years before the measurement of sCD163, which may have led to some degradation of the protein, although this has been proven very stable [12].

The SCORE risk estimation only gives estimates within the age 40 to 65 and some of our patients were younger than this; further, a substantial proportion of the patients could not have a SCORE risk estimated due to missing values of cholesterol and systolic blood pressure. This may conceale an underlying difference between the two groups.

The stratification of smoking status into smoking (ever) or non-smoking (never) carries no information on the amount of smoking exposure and causes a less certain matching. With > 95% of the patients in the smoking (ever) stratum the ability of extrapolating data to a non-smoking population is impeded.

Finally, the number of cases in our study was relatively low, and with a power of 80% to detect a difference of 0.5 mg/L sCD163, we cannot exclude the possibility of a statistical type II error overlooking significant changes.

## Conclusions

In this nested case-control study we did not find sCD163 to be a useful biomarker for prediction of first-time MI in a HIV-infected population.

## Competing interests

AK, GK, TB and AKj report no conflicts of interest.

HJM and Aarhus University have received royalties from IQ products, The Netherlands.

TLK has received research funding and/or honoraria from Bristol-Myers Squibb, Merck Sharp & Dohme, Glaxo Smith Kline, Abbott, Boehringer Ingelheim, Janssen-Cilag, Roche and Swedish Orphan.

JG has received funding from Abbott, Bristol-Myers Squibb, Merck Sharp & Dohme, Viiv, Gilead, Boehringer Ingelheim and Janssen-Cilag.

NO has received research funding from Roche, Bristol-Myers Squibb, Merck Sharp & Dohme, GlaxoSmithKline, Abbott, Boehringer Ingelheim, Janssen-Cilag, and Swedish Orphan.

AML has received research funding and/or honoraria from Abbott, Bristol-Myers Squibb, Gilead, Merck Sharp & Dohme, Glaxo Smith Kline, Boehringer Ingelheim and Janssen-Cilag.

## Authors’ contributions

AK: Analyzed and interpreted data and drafted the manuscript. HJM: Analyzed plasma samples and provided critical review of the manuscript. TLK: Contributed to conception and study design and provided critical review of the manuscript. JG: Contributed to conception and study design and provided critical review of the manuscript. NO: Head of the Danish HIV Cohort Study. Involved in analysis of data and provided critical review of the manuscript. GK: Contributed to conception and study design and provided critical review of the manuscript. TB: Contributed to conception and study design and provided critical review of the manuscript. Akj: Invovled in analysis of data and provided critical review of the manuscript. AML: Contributed to conception and study design and provided critical review of the manuscript. All authors read and approved the manuscript.

## Pre-publication history

The pre-publication history for this paper can be accessed here:

http://www.biomedcentral.com/1471-2334/13/230/prepub
